# 3D topographies promote macrophage M2d-Subset differentiation

**DOI:** 10.1016/j.mtbio.2023.100897

**Published:** 2023-12-06

**Authors:** Stefania C. Carrara, Amanda Davila-Lezama, Clément Cabriel, Erwin J.W. Berenschot, Silke Krol, J.G.E. Gardeniers, Ignacio Izeddin, Harald Kolmar, Arturo Susarrey-Arce

**Affiliations:** a Institute for Organic Chemistry and Biochemistry, Technical University of Darmstadt, Alarich-Weiss-Strasse 4, D-64287 Darmstadt, Germany; b Centre for Synthetic Biology, Technische Universität Darmstadt, Darmstadt, Germany; c Facultad de Ciencias de la Salud (FACISALUD), Universidad Autónoma de Baja California, Blvd. Universitario 1000, Valle de las Palmas, 22260 Tijuana, Mexico; d Mesoscale Chemical Systems, MESA+ Institute, University of Twente, P.O. Box 217, 7500AE Enschede, the Netherlands; e Institut Langevin, ESPCI Paris, CNRS, Université PSL, 75005 Paris, France; f Encytos B.V., Piet Heinstraat 12, Enschede, the Netherlands

**Keywords:** Structured surface, Fractal-like structures, Physical cues, Macrophage differentiation, anti-inflammatory macrophages, In vitro models

## Abstract

*In vitro* cellular models denote a crucial part of drug discovery programs as they aid in identifying successful drug candidates based on their initial efficacy and potency. While tremendous headway has been achieved in improving 2D and 3D culture techniques, there is still a need for physiologically relevant systems that can mimic or alter cellular responses without the addition of external biochemical stimuli. A way forward to alter cellular responses is using physical cues, like 3D topographical inorganic substrates, to differentiate macrophage-like cells. Herein, protein secretion and gene expression markers for various macrophage subsets cultivated on a 3D topographical substrate are investigated. The results show that macrophages differentiate into anti-inflammatory M2-type macrophages, secreting increased IL-10 levels compared to the controls. Remarkably, these macrophage cells are differentiated into the M2d subset, making up the main component of tumour-associated macrophages (TAMs), as measured by upregulated *Il-10* and *Vegf* mRNA. M2d subset differentiation is attributed to the topographical substrates with 3D fractal-like geometries arrayed over the surface, else primarily achieved by tumour-associated factors *in vivo*. From a broad perspective, this work paves the way for implementing 3D topographical inorganic surfaces for drug discovery programs, harnessing the advantages of *in vitro* assays without external stimulation and allowing the rapid characterisation of therapeutic modalities in physiologically relevant environments.

## Highlights

• M2d-subset macrophage is known to be the primary component of tumour-associated macrophages.
• Strategies to differentiate M2d-subset macrophages without needing a differentiation medium are demonstrated.
• 3D topographies induce M2d-Subset macrophage differentiation using the topography as physical cues.

## Graphical abstract

**Figure:**
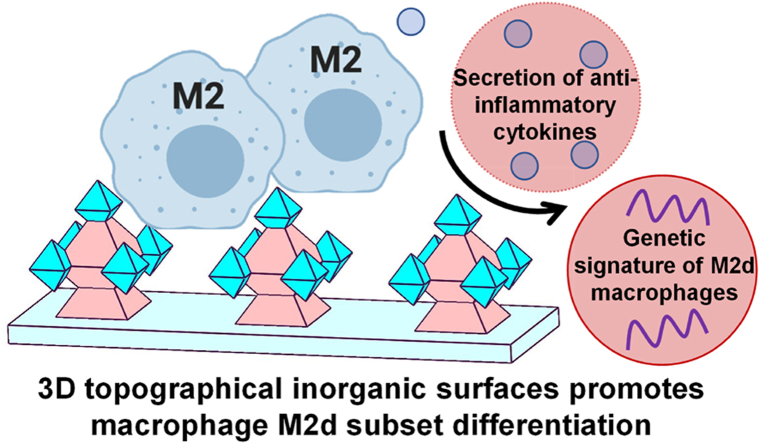

Drug discovery and personalised medicine have heavily relied on cellular models for the preclinical characterisation of potential drug candidates. The use of cell-based systems for drug discovery programs and their preferability over *in vivo* studies is primarily due to the time- and cost-savings associated with *in vitro* research. Unlike *in vivo* studies, they can be performed over the course of days, allowing higher reproducibility [1]. Nevertheless, these systems composed of two-dimensional (2D) cellular models often do not represent the entire picture once a potential drug proceeds into clinical development.

The question remains open on the type-to-cell model built for specific diseases. A potential avenue for drug screening is that the cellular system expresses the required cellular behaviour that mimics the disease. To close this gap, novel cellular systems are gaining attraction, such as 3D cultures [2,3] or organ-on-a-chip technology that brings higher physiological relevance [4]. While these appear to mimic physiological responses better than 2D cultures, they face several challenges. In the case of 3D cultures, issues with reproducibility and optimisation are often tackled, with high efforts required to optimise the culture conditions for every culture or a new cellular combination [5]. Organ-on-a-chip relies on microfluidic devices facing scalability issues for the microfabrication of devices to allow widespread use [4].

Topographical substrates can be tailored to unleash desirable cell behaviour and functions to overcome reproducibility and scalability challenges. A large number of replicas with high fidelity can be reproduced over several miniaturised well plates containing topographies [6]. The advantage of topographical surfaces relies on tailoring small geometrical designs to induce cellular growth or adherence, possibly mimicking *in vivo* conditions by using the topography as the physical cue. The geometrical designs can contain small features that cells use as stimuli. The stored information used as physical cues in such geometries could mimic natural substrates, such as extracellular matrix (ECM) proteins collagen, fibronectin, and laminin. These natural substrates are commonly used for cell cultivation, whereby cell surface receptors interact with the substrate and induce adhesion complex formation and possible cell proliferation or differentiation [7]. Synthetic substrates, such as poly-l-lysine, may also induce such responses *in vitro* [8].

A particular class of synthetic biomimetic inorganic topographical substrates, called fractal geometries, has been described based on naturally occurring geometries in several human body tissues [9]. Fractals are known for their high level of organisation, functional morphology, and similarity over a range of dimensional scales. These topographical architectures with increased hierarchy have successfully been used for the differentiation of intestinal tumour cells [10], as well as the formation of complex tumour spheroids [11]. Due to their multiscale geometry with a set of characteristic distances ranging from a few micrometers lattice spacing down to 500 nm features [12], fractal-like inorganic architectures arrayed over a substrate can serve as biomimetics, such as tissue and wound healing applications. In fact, the most significant representation of fractal geometries in the human tissues is the lungs [13,14], where lung-resident macrophages play a key role in regulating both injury and tissue repair [15]. Among these macrophages, two main populations exist: M1, which inhibits cell proliferation, and M2, which promotes cell proliferation and tissue repair. M1 or M2 macrophages exert pro- or anti-inflammatory functions, respectively, depending on their microenvironment [16]. Such pro- or anti-inflammatory functions have been observed for 3D structures with features like vertexes/cone angles (<60°), which are key for attachment and could promote pro- or anti-inflammatory cell polarisation, while triangular pyramids drastically reduce or even eliminate attachment [17]. Similar to fabricated 3D systems, the lung microenvironment limits the plasticity of alveolar-resident macrophages, resulting in M2 macrophages [18]. Within a tumour microenvironment, tumour-associated macrophages (TAMs) make up a significant part of M2 macrophages and regulate pro-tumour mechanisms by secreting anti-inflammatory cytokines, such as interleukin-10 (IL-10), resulting in increased tumorigenesis [19,20].

Highly defined micro- and nanopatterned surfaces have been developed to retain crucial *in vivo* functionalities, including cell proliferation and differentiation [21,22]. For several topographies, an upregulation of TAMs and cytokines have been reported [23,24]. Nonetheless, most of the focus has been on cellular reprogramming. Reprogramming M2 to M1 macrophages has been demonstrated to ameliorate the immunosuppressive tumour microenvironment [22]. Other reports revealed the possibility of inducing M2 responses by polarising macrophages with an upregulation of Arginase-1 induced by cytokines [25]. From previous reports, it is safe to say that physical stimuli induced by the topography can tip the balance toward a desired biological response. However, to our knowledge, M2d-subset differentiation has not yet been reported when using topographical designs.

Due to the inherent heterogeneity of macrophages, their *in vitro* characterisation and representation are not so trivial, despite several reports in literature [26,27]. In particular, the differentiation and classification of different subgroups has proven incredibly difficult using standard cell culture techniques. Hence, novel *in vitro* practices would provide an advantage to the field of drug discovery, particularly for defined macrophage polarisation. Therefore, as a proof of concept, this study used the monocytic THP-1 cell line to investigate the M2d-subset macrophage polarisation using fractal-like inorganic architectures arrayed over a surface. The approach demonstrated that THP-1 can differentiate using topographies of a few micrometers in size as a physical cue. Without the addition of external stimulus, these substrates could be used to investigate different therapeutic modalities in the field of tissue engineering and their modulation of tissue-specific macrophage differentiation using geometrical topographies. Compared to *in vivo* approaches, this provides a time-efficient and cost-effective alternative for drug discovery programs to characterise therapeutic modalities.

## 1 Results

**Fig. 1:**
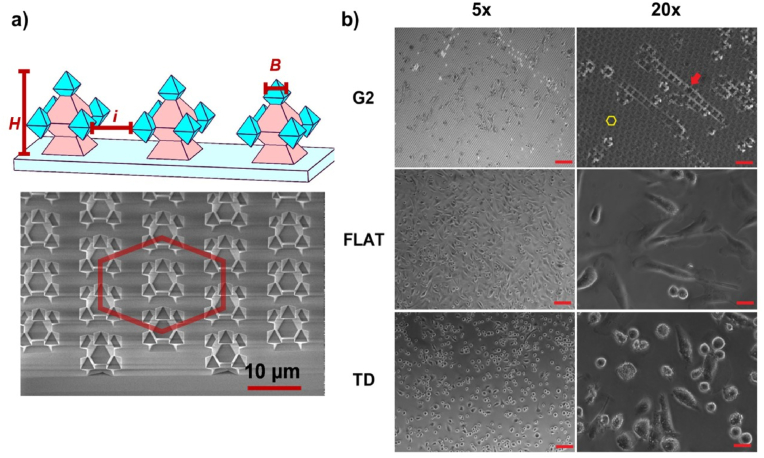
3D inorganic topographical substrates and cellular seeding. (a) G2 architectures depicting the interspace (*i*) of structure-to-structure nearest neighbour, the height (*H*), and end-octahedral diameter (*B*). The corresponding SEM image is shown below G2. The red hexagon represents the hexagonal (Hex) configuration of G2. (b) THP-1 macrophages on topographical substrates one-day post-seeding. The yellow hexagon represents the Hex configuration of G2. The red arrows highlight the different behaviour of THP-1 cells with the substrates. Scale bars represent 100 μm and 30 μm for 5x and 20× magnification, respectively.

*In vitro* models for complex cellular systems are required, mimicking the natural differentiation of cell lines. Particularly for immune cell subsets, their characterisation after *in vitro* differentiation through external stimulation depends on the supplemented source. Alternatively, a more reliable cellular system would result in the natural differentiation of cells based on the topography where cells reside. This is the case of inert silicon dioxide (SiO_2_) substrates decorated with fractal-like structures of varied morphology, i.e., from pyramids and octahedrons to fractals of increased hierarchy organised in periodic arrays. These arrays enable surface topography, which can be arrayed, adopting a hexagonal (Hex) configuration [11], as shown in Fig. 1, highlighted by the yellow arrow and hexagon.

The selection for these 3D structures is based on previous findings [11], where higher-order fractals such as second-generation (G2) foster the formation of cell layers, promoting spindle-like cell morphology with elongated nuclei [11]. Fig. 1a shows a schematic representation of G2 architectures. G2 has an interspace (*i*) of structure‐to‐structure nearest neighbour of 7 μm, height (H) of 10 μm, and end-octahedral diameter (*B*) of 2.5 μm. Flat SiO_2_ (FLAT) is used as a control. FLAT does not contain any fractal-like architecture. As an alternative control, regular sterile cell culture plastic without any coating is utilised, referred to as a tissue dish (TD).

To obtain M0 macrophages, monocytic THP-1 cells are primed with phorbol 12-myristate-13-acetate (PMA) for 24 h before recovering in complete growth media for 72 h. Between medium exchange, a PBS wash is performed. In the M0 macrophage state, the cells are seeded onto different substrates in a complete growth medium and cultivated over 14 days by partial replenishment with a fresh medium to ensure the cells remain viable. Observation of the cells under brightfield microscopy showed long, elongated macrophages over the 3D geometries, hinting at the particular differentiation of macrophages on the fractal structures versus FLAT and TD (Fig. 1b).

**Fig. 2:**
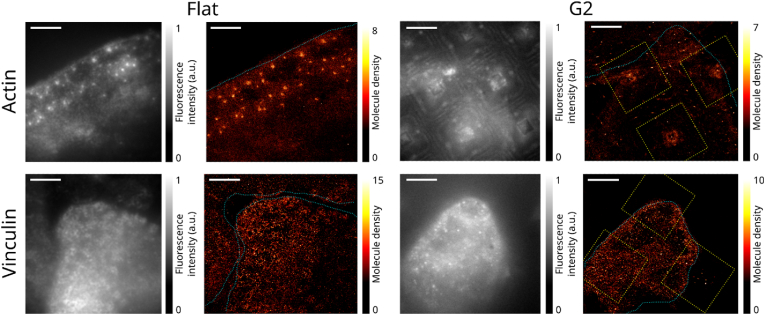
Diffraction-limited and super-resolution fluorescence images of actin and vinculin distribution in macrophages cultivated on flat or fractal substrates (G2). The samples were labelled against either actin or vinculin and imaged in dSTORM single molecule localisation microscopy. The top and bottom rows display the results obtained for actin and vinculin, respectively, after 4 days of cultivation. The left and right halves display the results on flat and fractal SiO_2_ substrates respectively. For each of the four conditions, the diffraction-limited and super-resolution images are displayed in gray and red-yellow respectively. Annotations in cyan represent the edges of the cell as determined by widefield diffraction-limited fluorescence microscopy acquired in the same areas, whereas the fractals are displayed in yellow dotted lines. Scale bars: 5 μm.

The morphology of substrate-differentiated macrophages is imaged with super-resolution fluorescence microscopy to investigate the spatial organisation of proteins. For this purpose, Single Molecule Localisation Microscopy (SMLM) is used, which relies on fluorescent immunolabelling of the proteins of interest with blinking probes attached to antibodies. Specifically, we used Direct Stochastic Optical Reconstruction Microscopy (dSTORM) [28,29], which relies on reversibly photo-switchable organic dyes to achieve photochemical blinking. The photo-switching allows to achieve a regime where the individual molecules are separated enough to be localized numerically, which returns the position of each fluorescent probe protein with a precision of around 10 nm. Fixed M2 macrophages are labelled with an organic dye (AlexaFluor647) targeting either vinculin or actin and imaged under single-molecule conditions thanks to dSTORM, similar to previously described [30]. Cells grown on FLAT substrates exhibit expected sizes and shapes, and the vinculin and actin organise in podosomes at adhesion sites (Fig. 2). More precisely, vinculin forms rings of 0.5–1 μm surrounding the central actin column [[30], [31], [32]]. On the other hand, cells grown on G2 fractal substrates appear very different. The vinculin does not organise in distinguishable ring-like patterns, and actin merely seems to follow the shape of the fractals without clustering in well-defined spots. These observations can be interpreted in two different ways - either M2 do not form podosomes when cultivated on G2 substrates, or they still form podosomes, but these have a strongly modified geometry and/or protein composition. In both cases, however, it is expected that such modifications of the podosome formation may induce significant changes in the adhesion, motility, and proliferation behaviour of macrophages at the cellular level. This correlates well with the different cell growth patterns observed between FLAT and G2 substrates in Fig. 1b.

**Fig. 3:**
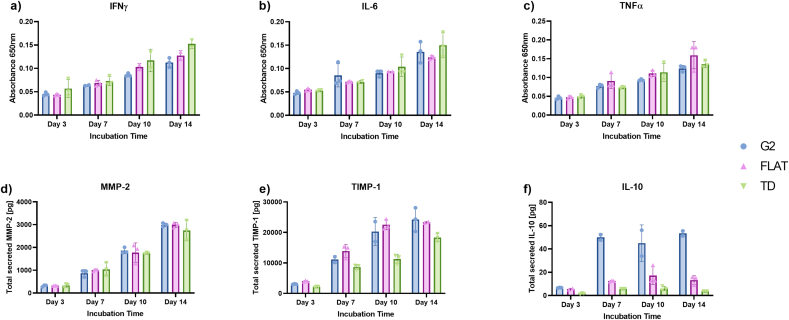
Analysis of protein secretion after 14 days in culture. M1 macrophage differentiation markers are measured using DuoSet ELISA assays (IFNγ, IL-6, TNFα) or MSD kits (MMP-2, TIMP-1). Secreted IL-10 is measured as an M2-specific macrophage marker. Error bars represent the standard deviation of biological replicates.

For further characterisation of macrophage-like cells, the seeded M0 THP-1 cells are incubated, and samples are collected from the supernatant after every 3–4 days. M1-and M2-specific macrophage markers are measured by ELISA or MSD assays (Fig. 3). For M1-specific markers, including IFNy, IL-6, TNFα, and MMP-2, no difference is observed between the 3D topographical substrate G2 and the FLAT or TD control (Fig. 3a–e).

To investigate M2-differentiation, IL-10 levels are determined (Fig. 3f). Seven days post-seeding, there was significantly increased of IL-10 after incubation on G2 substrates compared to FLAT, or TD. Due to biomimicry, the macrophage-like cells suggested a differentiation to an anti-inflammatory state, which aligns with, e.g., lung-resident macrophages and their role in tissue repair and healing.

**Fig. 4:**
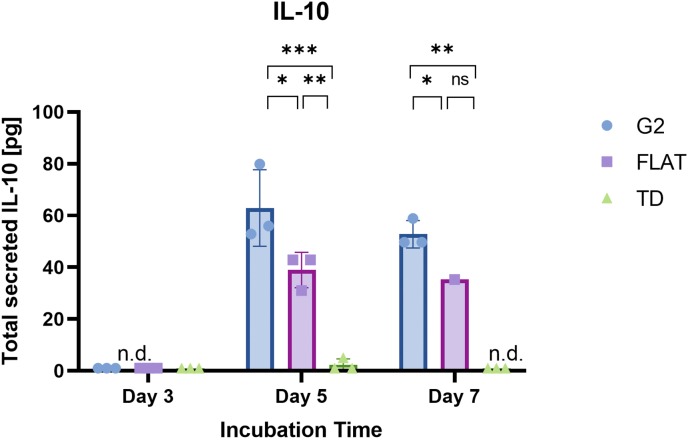
IL-10 secretion after 7 days incubation. Secreted IL-10 levels were measured after 3-, 5-, and 7-days incubation on either G2 (blue), FLAT (pink) or tissue dish (TD, green) by ELISA. Triplicate measurements were performed, and error bars show the standard deviation. n.d.: non-determined. One-way ANOVA analysis was performed: * - P ≤ 0.05 of G2 to FLAT.

To confirm the M2 differentiation, M0 macrophages are seeded on G2, FLAT or TD surfaces over 7 days, and samples are taken every 2–3 days. As shown in Fig. 4, Fig. 5 days of incubation without adding stimulants is sufficient to see a significant IL-10 increase in G2 over both FLAT and TD, with IL-10 levels under the detection limits at day 3. Interestingly, a longer incubation time did not result in higher IL-10 secretion beyond the levels expressed after 5 days in culture. This could presumably be because the cells have limited space to divide within the substrate area.

**Fig. 5:**
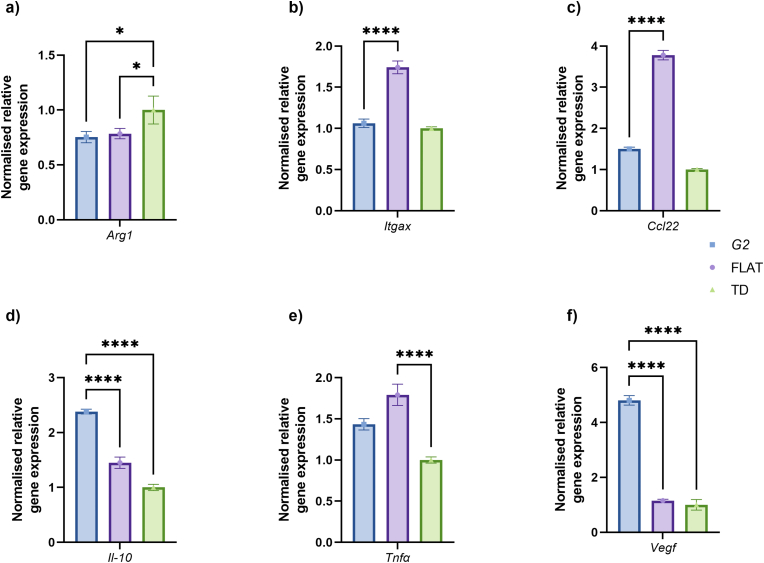
Gene expression analysis. RT-qPCR analysis performed 3 days after harvesting on either tissue dish (TD, green), flat (FLAT, purple) or G2 substrates (blue). The data is normalised using housekeeping genes *Gapdh* and *Hprt1*. The relative fold change is set to the tissue dish (TD) control. Triplicate measurements from 6 substrates were performed, and error bars show the standard error of the mean. One-way ANOVA analysis was performed: * - P ≤ 0.05, ****<0.0001.

With the information that M0 THP-1 macrophages differentiated themselves into an anti-inflammatory M2-like state on G2 substrates, gene expression analysis is performed to analyse specific M2 subsets. As described in the literature, four M2 subsets exist, referred to as M2a-d. For this, THP-1 macrophages are harvested three days post-seeding, RNA is isolated by pooling 6 substrates per isolation, and RT-qPCR is then performed using SYBR Green primers (see Materials). In the case of M2a subclass markers, no distinct Arg*1* upregulation was noted on the 3D topographical substrates compared to the controls (Fig. 5a). For *Itgax* and *Ccl22*, also M2a-specific markers, a very modest upregulation is seen for G2 compared to TD. However, the FLAT surface resulted in the highest upregulation (Fig. 5b–c). Lastly, the general M2 gene *Il-10* is verified, and as to be expected from the protein secretion results, *Il-*10 mRNA levels are significantly upregulated on G2 compared to the controls (Fig. 5d). Thus, the M2-differentiated cells appear to not belong to the M2a subgroup.

With defined gene signatures for other M2 subgroups of macrophage polarisation, the potential polarisation into M2b or M2d is assessed. Focusing on *Tnfα* for M2b, a similar pattern as M2a genes is observed, with the highest mRNA synthesis on the FLAT surface, followed by a very modest increase on G2 (Fig. 5e). This polarisation could be due to the interaction between the macrophages and the topographic substrate, as the cells appear to have a more elongated shape than on TD (Fig. 1). Lastly, relative *Vegf* mRNA levels are determined, where a 5-fold upregulation was observed upon incubation of the cells on the topographical substrates compared to the controls (Fig. 5f). VEGF and IL-10 are both associated with the M2d subdivision, indicating the specific polarisation of THP-1 macrophages to the M2d subtype after cultivation on G2 substrates without additional stimulants or inducers. With M2d macrophages representing the most predominant M2 subtype of tumour-associated macrophages, these results indicate the potential application of 3D inorganic topographical substrates for the development of physiologically relevant cellular systems for drug discovery programs.

## 2 Discussion

With an increasing need for novel *in vitro* cellular models that can mimic *in vivo* processes, great advances have been made to provide systems that better understand physiological processes. Within the field of tissue engineering, fractal-like topographies have the potential to serve as biomimetics of naturally occurring geometries within the human body, altering cellular responses in a natural approach. While much effort has been put into characterising different phenotypes and genetic signatures of *in vitro* models for macrophage polarisation, such as THP-1 cells or peripheral blood mononuclear cells (PBMCs) [33], their reproducibility and overlap with patient samples are often lacking. Similarly, other substrates have been used for the *in* vitro polarisation of immune cells with different surface chemistries or topographies [[34], [35], [36], [37]]. However, while most of these reported a cellular response towards the pro-healing M2 state, the herein presented study represents the first report, to the best of our knowledge, of a 3D topography resulting in a potentially specific M2d sub-polarisation of macrophages from a primed M0 state without the addition of further stimulants, as observed by the upregulation of M2-specific IL-10 at an mRNA and protein level, as well as the significant upregulation of *Vegf* mRNA after cultivation. The lack of external stimulus or inducers, such as IL-4 or IL-13, that are commonly added for M2 differentiation saves on reagents and does not force a certain pathway to be triggered but rather represents the natural process of polarisation of M2 macrophages *in vivo* after adhering and interacting with the 3D fractal structures. Future directions of this work would include in-depth immunophenotypic investigation (e.g., RNA sequencing) of different cellular states to provide further understanding of cellular differentiation and cell-to-substrate interactions.

The resulting naturally polarised tumour-associated macrophages can be used to investigate, e.g., re-polarisation strategies or efficacy studies of potential new drugs. Due to the generation of tumour-associated macrophages, cancer therapeutics can be assessed, with the vast potential of generating co-culture systems with these TAMs to build more complex biological systems. Furthermore, significant difficulties have been faced with the *in vitro* differentiation of macrophages into realistic TAM-like phenotypes, as intrinsic tumour factors are required. Additionally, the secretion of VEGF by M2d macrophages has a large implication in angiogenesis, a critical pathway for tumour progression [38,39]. When combined with tumour cells and/or other immune cells, a miniaturised tumour microenvironment could be reconstructed to characterise drug candidates in a physiologically relevant environment without needing *ex vivo* or patient-derived material. Hence, these substrates provide an attractive alternative thereof. Besides cancer therapeutics, these 3D substrates can be used to study cellular responses for wound-healing or autoimmune diseases.

The morphological study by SMLM resulted in different observations regarding podosome formation dependent on the cultivation surface. This observation correlates with the increased mRNA synthesis of *Itgax* only on FLAT compared to both G2 and TD. Integrin alpha X plays a role in the process of podosome formation and cellular motility [40], suggesting a relation between the topographical surfaces and the differentiation into M2d-like macrophages. Nonetheless, these observations require further investigation to understand how these podosome changes exert different cellular functions, including cellular adhesion and motility.

Compared to other materials, the inert characteristic of SiO_2_ may provide an advantage for other immune cell types or antigen-presenting cells (APCs). On other materials, these could react and secrete pro-inflammatory cytokines upon interaction with the surface, altering the cellular response of the cells [6]. Alternatively, the SiO_2_ might also increase macrophage phagocytosis rate, indicating M2 phenotype as they are better phagocytes. However, no detailed information about different M2 markers is presented [41]. Besides markers information, the material is not toxic for cellular growth, allowing cells to grow for up to 14 days with fresh medium supplementation. This attribute could be exploited in future co-culture studies, where cellular responses over a longer period are relevant. Furthermore, this includes prolonged exposure to chemicals for cell differentiation [10].

Interestingly, while most reported substrates utilised to drive cellular differentiation exhibit one or two given characteristic sizes (typically the spacing of the pattern or the width of the repeated feature), the fractal substrates show a multiscale set of characteristic distances ranging from 15 μm (lattice spacing) down to 500 nm, being the smallest fractal feature reported [12]. This could potentially be efficient in driving the differentiation towards cell fates that would otherwise be challenging to achieve. Lastly, the natural environmental cues that trigger macrophage polarisation of THP-1 cells could be expanded to other immortalised immune cell lines or primary cells (e.g., PBMCs) or healthy donor-derived macrophages to investigate their differentiation or assess phenotypic changes.

## 3 Conclusions

This study reports the natural polarisation of THP-1 macrophages without requiring any external stimulus by interacting with 3D topographic surfaces. The differentiation into the M2d subgroup makes this particularly interesting for drug discovery programs and the development of novel *in vitro* assays, facilitating cellular differentiation into subgroups that would otherwise be hard to achieve *in vitro*, even with the addition of reagents.

### 3.1 Materials

#### 3.1.1 Cell culture

THP-1 (DSMZ ACC 16) were cultured using a complete growth medium consisting of RPMI 1640 with 10 % foetal bovine serum (FBS). Cell maintenance was performed by sub-culturing every 2–3 days, maintaining a cell density of under 1 × 10^6^ cells/ml. For differentiation of monocytes to macrophages, cells were incubated with 200 ng/ml phorbol 1,3-myristate acetate (PMA) for 24 h at 37 °C in growth media. Thereafter, media was removed and cells were washed once with PBS. Subsequently, they were left to recover for 72 h in a growth medium before further handling. These cells are referred to as M0 THP-1 in this manuscript. All cell treatments were performed in T-75 (75 cm^2^) polystyrene cell culture flasks (Sarstedt).

#### 3.1.2 Substrates for cellular attachment

Throughout this study, three different substrate types were utilised for cell attachment and cultivation. Fractals described, a glass control (FLAT) and lastly generic plastic dishes (tissue dish = TD). The tissue dish was a tissue culture-treated polystyrene plate (Corning 3526). Fractals and glass substrates were placed in a 24-well tissue culture-treated plate (Corning 3526) and sterilised by irradiation for 1 h before proceeding with cell seeding described below.

#### 3.1.3 Seeding of cells on substrates

Following irradiation of the substrates, complete growth medium was removed from the M0 THP-1 cells in a T-75 flask. Cells were then rinsed with PBS and detached using Trypsin. After resuspending, 50 μl M0 THP-1 cell suspension at 4 × 10^5^ cells/ml were seeded onto the substrates in 24-well plates to ensure homogeneous distribution on the surface. Once the cells became adherent, approximately 3–4 h after seeding, 1 ml RPMI 1640 with 10 % FBS was added. The cells were incubated at 37 °C, 5 % CO_2_, until harvested. The medium was replenished for longer experiments by removing and adding 250 μl fresh medium to ensure the cells remained viable.

#### 3.1.4 Super-resolution acquisitions sample preparation

Cells were fixed four days post-seeding in a solution containing 4 % paraformaldehyde (PFA) and 0.2 % glutaraldehyde in PBS, at 37 °C for 10 min, followed by three rinses in PBS. Cells were permeabilised with PBS containing 0.1 % Triton X-100 for 10 min and rinsed three times with PBS. Following, cells were reduced with NaBH_4_ at 1 mg/ml for 10 min twice and rinsed three times with PBS. Then the cells were labelled against either vinculin or actin.

For vinculin labelling, t cells were incubated for 30 min in PBS +1 % BSA for the labelling, then for 1 h at 37 °C with 1:300 mouse anti-vinculin antibody (Sigma Aldrich, V9131) in PBS + 1 % BSA. This was followed by three washing steps in PBS +1 % BSA, incubation overnight at 4 °C with 1:300 goat anti-mouse AF647 antibody (Life Technologies, A21237) diluted in PBS + 1 % BSA and three more PBS washes. Finally, the cells were post-fixed with 3.6 % formaldehyde for 15 min in PBS. The cells were washed in PBS three times and then reduced for 10 min with 50 mM NH_4_Cl, followed by three additional washes in PBS. Samples were stored at 4 °C until imaging.

For actin, labelling was performed just before acquisition according to the following protocol: Cells were incubated at room temperature for 15 min with phalloidin-conjugated AlexaFluor647 (Life Technologies, A22287) at a concentration of 20 nM in the dSTORM Imaging buffer (see section Super-resolution microscopy and acquisition). The acquisition was performed immediately after imaging without further rinsing.

#### 3.1.5 Super-resolution microscopy and acquisition

We used a custom-built inverted microscope with an RM21 body and a MANNZ micro- and nano-positioner (Mad City Labs). The illumination and fluorescence collection were done with a Nikon 100x 1.49NA APO TIRF SR oil immersion objective. The excitation was performed with a 638 nm laser (LBX-638-180, 180 mW, Oxxius) with a 405 nm laser for pumping (LBX-405-50, 50 mW, Oxxius). A full multiband filter set (LF405/488/561/635-A-000, Semrock) was used. The excitation consisted of a standard vertical Gaussian beam. The fluorescence was recorded on the EMCCD camera (iXon Ultra 897, Andor) with a pixel size of 107 nm in the object plane.

EMCCD acquisitions were made with a 30 ms exposure time and a gain of 100. We used a dSTORM buffer composed of 100 mg/ml glucose, 3.86 mg/ml MEA, 0.5 mg/ml glucose oxidase and 1.18 μl/ml catalase in PBS and a 638 nm continuous excitation with an irradiance of 5 kW/cm^2^. Data was acquired for 20 min for each region of interest. A low power (0.01 kW/cm^2^) continuous 405 nm excitation was also added to increase the density of detections.

Since the thickness does not allow imaging through the fractal substrates due to the low working distance of the objective, the substrates were flipped down and deposited on a round 25 mm diameter coverslip (170 μm thickness) with a 50 μl imaging buffer between the two substrates. For each condition, datasets were acquired on several fields of view, with an average of 3 fields of view per condition. The most representative images were displayed in Fig. 2.

#### 3.1.6 Protein secretion

For the determination of protein secretion, samples from the supernatant were taken at the indicated time points after cell seeding. For longer incubations (e.g., 14 days) where medium exchange was required, 250 μl were removed every 4 days, collected and replaced with fresh medium. For data analysis, the dilution factor was considered when calculating the total secreted protein. Experiments were performed in at least three independent runs in biological triplicates. The following kits were used for protein determination following the respective manufacturer's instructions: Human IL-10 DuoSet ELISA (R&D Systems), Human TNFα DuoSet ELISA (R&D Systems), Human IFNγ DuoSet ELISA (R&D Systems), Human IL-6 DuoSet ELISA (R&D Systems), R-PLEX Human MMP-2 Antibody Set (Mesoscale Discovery), Human TIMP-1 Kit (Mesoscale Discovery). One-way ANOVA was performed between different substrate groups using GraphPad Prism 8.0.

#### 3.1.7 Gene expression analysis

Three days post-seeding, RNA isolation was performed using the RNeasy Micro kit (QIAGEN). To ensure sufficient RNA was isolated, 6 substrates were pooled for each treatment condition. Following RNA isolation, RT-qPCR was performed using iTaq Universal SYBR Green One-step Kit and pre-designed SYBR green primers from Sigma Aldrich. Detection was performed using a CFX Connect Real-Time PCR machine (Bio-Rad) in triplicates. Gene expression analysis was determined by normalising the data to housekeeping genes *Gapdh* and *Hprt1* and setting the data relative to the tissue dish control using the CFX Manager software (Bio-Rad). Visualization was performed using GraphPad Prism 8.0, depicting the standard error of the mean. SYBR Green primer sequences (all represent human genes):

*Gapdh* FWD 5′-ACATCGCTCAGACACCATG-3’

*Gapdh* REV 5′-TGTAGTTGAGGTCAATGAAGGG-3′,

*Ccl22* FWD 5′-GTGGTGTTGCTAACCTTC-3’

*Ccl2*2 REV 5′-GGCTCAGCTTATTGAGAATC-3′,

*Il-10* FWD 5′-GCCTTTAATAAGCTCCAAGAG-3’

*Il-*10 REV 5′-ATCTTCATTGTCATGTAGGC-3′,

Arg*1* FWD 5′-GGTGACTCCCTGTATATCTG-3’

Arg*1* REV 5′-TTCTTCCTAGTAGATAGCTGAG-3′,

*Itgax* FWD 5′-GCCTGGATTATAAGGATGTC-3’

*Itgax* REV 5′-TTGAAAAGCTAATCCAACCC-3′,

*Vegf* FWD 5′- TGCAGATTATGCGGATCAAACC-3’

*Vegf* REV 5′- TGCATTCACATTTGTTGTGCTGTAG-3′,

*Tnfa* FWD 5′- CTCTTCTGCCTGCTGCACTTTG-3’

*Tnfa* REV 5′- ATGGGCTACAGGCTTGTCACTC-3’

## Author contributions

The manuscript was written through the contributions of all authors. All authors have approved the final version of the manuscript.

**Stefania C. Carrara:** Writing – original draft, Supervision, Conceptualization. **Amanda Davila-Lezama:** Validation, Investigation. **Clément Cabriel:** Writing – original draft, Methodology, Investigation, Formal analysis. **Erwin J. W. Berenschot:** Validation, Investigation. **Silke Krol:** Writing – review & editing, Methodology. **J.G.E. Gardeniers:** Writing – review & editing, Resources. **Ignacio Izeddin:** Writing – review & editing, Resources. **Harald Kolmar**: Writing – review & editing, Resources. **Arturo Susarrey-Arce:** Writing - review & editing, Visualization, Resources, Project administration.

## Declaration of competing interest

The authors declare that they have no known competing financial interests or personal relationships that could have appeared to influence the work reported in this paper.

## Data Availability

Data will be made available on request.
